# Supplementary material for: Frequency and quality of first aid offered by older adolescents: a cluster randomised crossover trial of school-based first aid courses
Source: PeerJ. 2020 Aug 17;8:e9782. doi: 10.7717/peerj.9782 (PMC7439956; doi:10.7717/peerj.9782)
Supplement: Supplemental Information 4 [file peerj-08-9782-s004.docx]

**Scoring Criteria for Open-Ended Physical Health First Aid Responses - what did respondent do to help person with physical injury or emergency**

|  | **Component of physical health first aid response** | **0 point (no mention or inadequate response)** | **1 point (detailed)** |
| --- | --- | --- | --- |
| 1 | DRSABCD (S1) | No mention on how to approach person   - No correct references to DRSABCD or response unrelated to DRSABCD - Non-descriptive | Respondents noted that they used the “DRSABCD” acronym in their response   - Situation is related to using DRSABCD |
| 2 | D – Danger (S1) | No mention on how to approach person | Responses could include:   - Checking surroundings and ensure safety (out of danger) |
| 3 | R - Response (S1) | No mention on how to approach person or unrelated response  Example response: “Talk to them and just listen to what they're going through” or “ask if ok” | Responses could include:   - Check for consciousness - Talk and touch method: attempt to talk to person, touch hands or squeeze shoulder |
| 4 | S - Send for help (S1) | No mention on how to approach person   - Take person to sick bay or call an adult/teacher/nurse   Example response: ”Help them find an adult or a full trained first aid person” or “I called for help” | Responses could include:   - Calling 000 for help - Call ambulance/emergency services |
| 5 | A – check airway (S1) | No mention on how to approach person | Responses could include:   - Check if person is responding and their airway is clear - If person is unconscious, keep airway open and clear (of foreign objects), tilt head gently and check for breathing - If person’s mouth is obstructed, put person in the recovery position (positing a person to their side) and attempt to clear mouth |
| 6 | B – check breathing (S1) | No mention on how to approach person | Responses could include:   - Check chest movements - Listen to sounds from mouth and nose |
| 7 | C – begin CPR (S1) | No mention on how to approach person | Responses could include:   - Stating CPR was used - Performing compressions |
| 8 | D – automated external defibrillator (AED; S1) | No mention on how to approach person | Responses could include:   - Following instructions on the AED machine and performing them until ambulance arrives |
| 9 | Sprains and strains (S2) | No mention on how to approach person   - Response does not include minimum reference to ice and at least one of the other ways to manage injury | Responses could include:   - RICER = Rest, Ice, Compression, Elevation, and referral - Rest – rest and avoid activities that causes considerable pain - Ice – 15 to 20 minutes every one to two hours - Compression - (apply firm bandages that does not restrict circulation or additional pain; covering whole joint) - Elevation – elevate limb - Referral – have them seek health professional or emergency services - Response can say RICER along another component of acronym |
| 10 | Fractures and dislocations (S2) | No mention on how to approach person | Responses could include:   - Provide bandaging or slings - Provide a comfortable position for person - Monitor for shock - Seek medical assistance - Do not relocate or realign fracture - Use ice for injury (if needed) |
| 11 | Wound care (S2) | No mention on how to approach person | Responses could include:   - Stop/reduce bleeding with appropriate procedures, such as using bandage - Cover would with sterile dressing - Apply pressure over wound using a pad - Check circulation below wound - Use tourniquet to clot bleeding (if applicable) - Cleaning or sterilizing wound with appropriate materials (e.g saline, antibacterial cream) |
| 12 | Shock (S2) | No mention on how to approach person | Responses could include:   - Person was in shock, I called 000 for help - ensure person has clear airway and breathing normally - help with circulation is recommended – lie or sit person down and elevate legs whenever possible - control external bleeding (if applicable) |
| 13 | Concussion (S2) | No mention on how to approach person | Responses could include:   - Check for alertness, responsiveness, and talking - Do cold compression on concussed area. - Check and control bleeding, cover wounds (if needed) - If unconscious, follow DRSABCD |
| 14 | Asthma (S2) | No mention on how to approach person | Responses could include:   - Providing assistance with asthma bronchodilator (puffer) and spacer (and do not leave person) - If no improvement, call emergency services |
| 15 | Diabetes (S3) | No mention on how to approach person | Responses could include:   - If unconscious, support person on their side and call ambulance - Giving person sugar, like chocolate - Give them sandwich or several sweets if person is more alert |
| 16 | Allergies (S3) | No mention on how to approach person | Responses could include:   - It differs depending on type of allergy; provide medication, use epi pen, and/or call 000 |
| 17 | Anaphylaxis (S3) | No mention on how to approach person | Responses could include:   - Providing assistance with adrenaline autoinjector (EpiPen) - If breathing is difficult, lay person flat; do not let them walk - Call ambulance/emergency services - Commence CPR if person is unresponsive and not breathing normally |
| 18 | Poisons (S3) | No mention on how to approach person   - Induce vomiting | Responses could include:   - Call 000 or Poisons Information Centre (131126) and get advice on what to do |
| 19 | Exposure to heat (S3) | No mention on how to approach person | Responses could include:   - Apply cold packs to neck, groin, and armpits - Giver water to drink if fully conscious - Move person to a cool place with circulating air - Loosen tight clothing or unnecessary garment - Lie the person down - Follow DRSABCD if necessary |
| 20 | Exposure to cold (S3) | No mention on how to approach person   - Any mentions of rubbing or exposing limbs to radiant heat | Responses could include:   - Find warm shelter - Put on dry clothes if person is wet - Have the person drink warm fluids; and move around - Warm up under a blanket - If fingers and hands are cold, have person put them into their armpit |
| 21 | Seizures (S3) | No mention on how to approach person | Responses could include:   - Protect injury of head (e.g., put flat jacket under head) and put person in recovery position - Do not put anything in mouth or attempt to restrain person or stop jerking - Stay with person and call 000/ambulance - Talk to person until they regain consciousness |
| 22 | Spinal injuries (not covered in training) | No mention on how to approach person | Reponses could include:   - Keeping patient still to avoid further injury - Move patient out of danger/to safe area using extreme care - If unconscious, follow DRSABCD |
| 23 | Fainting (not covered in training) | No mention on how to approach person | Responses could include:   - If unconscious, follow DRSABCD - Raise legs above chest height - Treat any injuries from fall |
| 24 | Burns (not covered in training) | No mention on how to approach person   - Mentions applying cream or oil to burn - Mentions applying ice to burn - Trying to break blisters | Responses could include:   - Cool the area by applying cool running water - Apply a sterile dressing |

Updated 12.04.2018 CJO
