# Supplementary material for: Frequency and quality of first aid offered by older adolescents: a cluster randomised crossover trial of school-based first aid courses
Source: PeerJ. 2020 Aug 17;8:e9782. doi: 10.7717/peerj.9782 (PMC7439956; doi:10.7717/peerj.9782)
Supplement: Supplemental Information 5 [file peerj-08-9782-s005.docx]

**Coding frame for teenAID help given to peer with an injury/emergency question (physical first aid) –**

**“What was the reason(s) that you were not able to help the person?”**

| # | Code | Example | Notes |
| --- | --- | --- | --- |
| 1 | No help needed | - “no one need help” - “There was no one to help.” - “wasn't there and he didn't require immediate first aid” | This differs from 3: ‘No previous encounters’ in that there was a person with a problem, but help did not appear necessary |
| 2 | Someone else provided help | - “already being helped by a big group of people” - “someone else was taking care of them” - “There was already a trained doctor on the scene” |  |
| 3 | No prior encounters | - “because there was no one that I knew of who had an accident” - “i have not come across anyone with a physical injury that required first aid.” - “i didn’t see anyone with a problem” |  |
| 4 | Don’t know/unsure | - “Not sure” - “I don’t know” | Respondent doesn’t know or is unsure of *why they didn’t help*. Don’t include responses that are unsure of *what to do*. These should be coded under 7: Lack adequate skills/experience |
| 5 | Unaware that first aid was needed | - “I did not know about it.” - “didn't realize/understand the problem” |  |
| 6 | Negative Perspective | - “TOO WEIRDED OUT” - “CAUSE I DONT LIKE HIM” |  |
| 7 | Lack adequate skills/experience | - “I don’t know how to” - “Because I am not confident enough to do first aid” - “because I was too shy and scared to do it” |  |
| 8 | No established rapport | - “didn't know that person” - “didn’t trust the person” |  |
| 9 | Practical Constraints | - “I wasn't there when it happened and was unable to help at the time.” - “because I wasn't there when it happened” | Various circumstances prevented bystander from helping, including lack of time and physical distance |
| 10 | No reason | - “middle of a footy game - “no reasons” | The respondent provided a response but did not indicate why they did not help the person in need of care |
| 11 | No appropriate code available | - “nothing” - “because” - “Myself” | Responses did not relate to the question, incoherent or they were not complete ideas |

**Coding frame for teenAID help given to peer with a mental health problem/crisis question –**

**“What was the reason(s) that you were not able to help the person?”**

|  | **Code** | **Includes/examples** | **Notes** |
| --- | --- | --- | --- |
| 1 | No prior encounters | - “Because I have not seen anyone with a mental problem in the area I live in.” - “because there was nobody to help” - “They don’t exist” | Respondents may have been prompted to answer the question but should not have been |
| 2 | No established rapport | - “because i dont know them to well” - “Becuase I didn't know her” - “didn't know the person” |  |
| 3 | Someone else provided help | - “because other people were able to help them better than I could” - “because that person already is having help” - “He had other closer friends who were helping this person.” |  |
| 4 | No help needed | - “Because they didn't need help” - “It was not a condition that was detrimental to their health or wellbeing.” - “Because they didn’t talk to me about it” | This differs from 1: ‘No previous encounters’ in that there was a person with a problem, but help did not appear necessary |
| 5 | Minding my own business/Did not want to interfere | - “I did not want to interfere.” - “I did not want to get involved in their personal life” - “minding my own business**”** |  |
| 6 | Unaware that MHP occurred | - “Did not know of the problem at the time” - “I didn't know if they needed help or not” - “i didn’t know they had mental health issues” |  |
| 7 | Lack adequate skills/experience | - “I am not experienced” - ‘I did not know what to do/say to make them feel any better” - “I didn't feel qualified and didn't want to make things worse in case I did do something wrong.” |  |
| 8 | Don’t know/unsure | - “I don't know” - “not sure” | Respondent doesn’t know or is unsure of *why they didn’t help*. Don’t include responses that are unsure of *what to do*. These should be coded under 7: Lack adequate skills/experience |
| 9 | Negative perspective | - “he is going to attack me if I talk to him” - “He scared me.” - “I was scared because he was in a very arrogant mood.” | This includes any form of stigma |
| 10 | Practical constraints | - “I have mental health too” - “Didn't have the time” - “wasn't there when it happened” | Various circumstances prevented bystander from helping, including lack of time and physical distance |
| 11 | I did help | - “I did help ok” - “I knew how to help her but all needed to do to help her was just talk to her and help her through some of the problems” |  |
| 12 | I did not want to help | - “Didn't feel like” - “I’m lazy and could not be bothered getting up” | Respondent indicated that they did not want to help the person with MHP |
| 13 | No Reason | - “Because I couldn't” - “He seemed like he was under the effects of drugs.” | The respondent provided a response but did not indicate why they did not help the person in need of care |
| 14 | No appropriate code available | - “because yolo” - “Aptitude” | Responses did not relate to the question, incoherent or they were not complete ideas |
