# Supplementary material for: Frequency and quality of first aid offered by older adolescents: a cluster randomised crossover trial of school-based first aid courses
Source: PeerJ. 2020 Aug 17;8:e9782. doi: 10.7717/peerj.9782 (PMC7439956; doi:10.7717/peerj.9782)
Supplement: Supplemental Information 8 [file peerj-08-9782-s008.pdf]

teenAID - Participant information (P3)

What is First Aid Training for Common Injuries or Emergencies in Teens?
The "First Aid Training for Common Injuries or Emergencies in Teens" program is a 3 hour non-accredited course, designed to equip adolescents with the skills to provide basic physical first aid to their peers. The program is designed to develop students' knowledge and skills in:
• Chain of survival
• DRSABCD
• Recovery position
• CPR and defibrillation
• Basic first aid for common injuries and emergencies such as sprains, strains, fractures, dislocations, concussions, asthma, anaphylaxis etc.

The program is presented by an experienced first aid instructor from Australian Red Cross. Teaching sessions involve a powerpoint presentation and practical demonstrations of techniques such as the recovery position and CPR.

What is the purpose of this research?
The research aims to compare two different types of first aid courses for teens ("teen Mental Health First Aid", and "First Aid for Common Injuries and Emergencies in Teens"), to assess their effectiveness in teaching teens to assist their peers in a first aid situation.

Who is conducting this research?
This research is being conducted by a team from the University of Melbourne. Funding for this program has been provided by an Australian Rotary Health Research Project Grant.

Two of the investigators are employees of the not-for-profit organisation Mental Health First Aid Australia and another is chair of its board.

What will the research involve for students?
To track how students' knowledge of physical injuries and first aid strategies change over time, students will be asked to complete questionnaires at three time points: before the program is presented, after the teaching session/s, and 12 months after the training has finished. The questionnaires will be completed online and it is estimated they will each take around 20-30 minutes to complete. Students will be able to complete the questionnaires during class time.

Is participation in this research confidential?
The program will be presented during students' normal class times. This means that student identities cannot be kept completely anonymous from the program instructor or the research manager who will be present. However, all information provided when responding to the questionnaires is confidential. All results from the questionnaires will be published in the form of group percentages and not individual responses. The information provided by participants will be stored electronically and held under password protection, for a minimum period of 5 years. The data will not be used for any purposes other than those described here.

According to the preference of the school, student surveys will either be sent by the school to the student's email address, without any contact details being given to the research team, or will be handed to students in hard copy during class time. Alternatively, students can select in the second questionnaire to receive the following questionnaires directly to their personal email address by supplying this to the research team. This option is, however, the individual preference of the student and there are no repercussions if an email address is not supplied.

Names will not be used on the questionnaire and a Victorian Student Identification Number will be used in their place. The use of student ID numbers is considered necessary by the research team to ensure the safety of participants. In the event of a participant requesting assistance as a result of becoming distressed, the student ID allows the research team to notify the school of the student's identity without providing the identity to the research team. Student ID numbers will be removed from files storing survey responses, but a key will be retained linking survey IDs with student IDs so that we can match surveys across time points. Data will be stored with survey IDs, with the linking key stored securely in a separate file.

While every effort will be made to protect the identity of participants, there are legal limitations to data confidentiality. For instance, it is possible for data to be subject to subpoena or freedom of information request. However, all data will be retained in a de-identified format.

How will the outcome/results of this research be made public?
The findings of this research will be published in a scientific journal, on the mental health first aid website (www.mhfa.com.au) and in training materials. Results may also be presented and discussed at local, national and international conferences on youth mental health, or mental health first aid.

Are there any risks?
The questionnaires contain some questions about mental illness, which can be a little distressing to some people. A number of resources, such as helplines and websites, will be given to students before and after the training. A welfare coordinator will be present at times when student surveys are being completed.

If you are distressed about the issues discussed in the training or in the questionnaires, there are a number of services that are available for you to contact, provided at the end of this questionnaire. Alternatively, you can contact the research assistant, Ms. Alyssia Rossetto, on alyssia.rossetto@unimelb.edu.au

What are the benefits?
Students will receive the training free of charge. By attending this course, students will be taught a range of skills for assisting others in the event of a physical injury.

The information provided in the questionnaires will assist us in developing first aid materials in the future.
Participation in this research will therefore benefit future students across Australia and internationally, who receive the training.

How can I get further information?

If you would like further information before deciding whether to participate, please contact:
Ms. Alyssia Rossetto
email: alyssia.rossetto@unimelb.edu.au, phone: 03 9035 6029
Population Mental Health Group, University of Melbourne
Alyssia is the Research Assistant for the teenAID evaluation project.

If you have questions about the first aid training or first aid instructors, please contact the Research Assistant, Ms. Alyssia Rossetto, who can forward on your query to St John Ambulance Australia.

Laura Hart is the Chief Investigator of the teenAID evaluation project, and is responsible for the design and conduct of the trial.
email: lhart@unimelb.edu.au
Population Mental Health Group, University of Melbourne

If you have questions about the scientific aspects of the study, please contact:
Professor Tony Jorm
email: ajorm@unimelb.edu.au, phone: 03 9035 7799
Population Mental Health Group, University of Melbourne
Tony is a Professorial Fellow and Scientific Director of MHFA Australia.

This research has been approved by University of Melbourne Human Research Ethics Committee.
If you have concerns about the ethics of the study, please contact:
Manager
Human Research Ethics, The University of Melbourne
phone: 03 8344 2073
Ethics approval number: 1341238.3
Version 2 May 2014

teenAID

This is about whether you agree to participate in the research.

1. Please enter your Participant Identification Number

2. **Would you like to proceed with the survey?**

- ◯ Yes, I have read and understood the information provided to me and would like to proceed
- ◯ No, I do not want to complete this survey

teenAID - John's story

Please read John's story carefully, as the following questions relate to it.

John's Story
John is a 16 year old who has been unusually sad and miserable for the last few weeks. He is tired all the time and has trouble sleeping at night. John doesn’t feel like eating and has lost weight. He can’t keep his mind on his studies and his marks have dropped. He puts off making any decisions and even day-to-day tasks seem too much for him. His parents and friends are very concerned about him. John says he will never be happy again and believes his family would be better off without him. John says he feels so desperate, he has been thinking of ways to end his life.

3. What, if anything, do you think is wrong with John?
Please write your answer in the box

4. If John was a friend, I would help him.

Strongly disagree	Disagree	Somewhat disagree	Neither agree or disagree	Somewhat agree	Agree	Strongly agree
◯	◯	◯	◯	◯	◯	◯

5. If John was a friend, I would...

Please note: you can select more than one option.

- [ ] Tell John what he needs to do to fix his problems.
- [ ] Invite John to hang out and do something fun with me.
- [ ] Ignore John because he is being attention-seeking.
- [ ] Let John know I won't want to be friends with him any more if he's like this all the time.
- [ ] Avoid talking about suicide, because it might put the idea in John's head.
- [ ] Tell John I have noticed something seems wrong, and I want to make sure he is okay.
- [ ] Suggest John tell a health professional about his problems (e.g. a counsellor, GP, or psychologist).
- [ ] Suggest John tell an adult (other than a health professional) about his problems (e.g. a parent or teacher).
- [ ] Ask John if he is thinking of suicide.
- [ ] Encourage John to take responsibility and deal with his problems on his own.
- [ ] Listen to John talk about his problems.
- [ ] Not do anything.

Other (please specify):

6. If John was a friend, how confident would you feel in helping him?

Not at all confident	A little bit confident	Moderately confident	Quite a bit confident	Extremely confident
○	○	○	○	○

7. Which of the following people do you think would be helpful, harmful, or neither helpful nor harmful, for John’s problem?

	Helpful	Neither	Harmful
Counsellor	○	○	○
General practitioner (GP) or family doctor	○	○	○
Psychologist	○	○	○
Close friend	○	○	○
Family member	○	○	○
Parent	○	○	○
Teacher	○	○	○
School Welfare Coordinator/School Counsellor	○	○	○
Minister or priest	○	○	○

8. In the list provided in the question above, which one person do you think is the most helpful for John's problem?

- ◯ Close friend
- ◯ Counsellor
- ◯ Family member
- ◯ General practitioner (GP) or family doctor
- ◯ Minister or priest
- ◯ Parent
- ◯ Psychologist
- ◯ School Welfare Coordinator/School Counsellor
- ◯ Teacher
- ◯ None of the above

9. If I had a problem right now like John's, I would...
Please note: you can select more than one option.

- ☐ Try to deal with it on my own.
- ☐ Talk to a friend about it.
- ☐ Talk to a health professional about it (e.g. a counsellor, GP or psychologist).
- ☐ Talk to an adult (other than a health professional) about it (e.g. a parent or teacher).
- ☐ Improve my diet.
- ☐ Try to get more sleep.
- ☐ Spend more time with friends.
- ☐ Try to do more exercise.
- ☐ Look for information about my problem on the internet.
- ☐ Do nothing.

Other (please specify):

10. Please indicate how strongly you personally agree or disagree with each statement

	Strongly disagree	Disagree	Neither agree nor disagree	Agree	Strongly agree
People with a problem like John could snap out of it if they wanted.	◯	◯	◯	◯	◯
A problem like John's is a sign of personal weakness.	◯	◯	◯	◯	◯
John's problem is not a real medical illness.	◯	◯	◯	◯	◯
People with a problem like John's are dangerous.	◯	◯	◯	◯	◯
It is best to avoid people with a problem like John's so that you don't develop this problem.	◯	◯	◯	◯	◯
People with a problem like John's are unpredictable.	◯	◯	◯	◯	◯
If I had a problem like John's I would not tell anyone.	◯	◯	◯	◯	◯

11. The following questions ask how you would feel about spending time with John.
Would you be happy to:

	Yes definitely	Yes probably	Probably not	Definitely not
Go out with John on the weekend?	◯	◯	◯	◯
Work on a project with John?	◯	◯	◯	◯
Invite John around to your house?	◯	◯	◯	◯
Go to John's house?	◯	◯	◯	◯
Develop a close friendship with John?	◯	◯	◯	◯

teenAID - Jeanie's story

Please read Jeanie's story carefully, as the following questions relate to it.

Jeanie's Story
Jeanie is a 16 year old living at home with her parents. Jeanie started at your school last year and you are the only friend she has made so far. She seems very shy and when you ask her why she doesn't make more of an effort, she says she would really like to make more friends but is scared that she'll do or say something embarrassing when she's around others. Although Jeanie's schoolwork is OK she rarely says a word in class and becomes incredibly nervous, trembles, blushes and seems like she might vomit if she has to answer a question or speak in front of the class. At her house you have seen that Jeanie is quite talkative with her family, but becomes quiet if anyone she doesn't know well comes over. She has stopped answering the phone and doesn't come to parties anymore. Jeanie says she knows her fears are unreasonable but she can't seem to control them and this really upsets her.

12. What, if anything, do you think is wrong with Jeanie?

13. If Jeanie was a friend, I would help her.

Strongly disagree	Disagree	Somewhat disagree	Neither agree or disagree	Somewhat agree	Agree	Strongly agree
○	○	○	○	○	○	○

14. If Jeanie was a friend, I would...
Please note: you can select more than one option.

- ☐ Tell Jeanie what she needs to do to fix her problems.
- ☐ Invite Jeanie to hang out and do something fun with me.
- ☐ Ignore Jeanie because she is being attention-seeking.
- ☐ Let Jeanie know I won't want to be friends with her any more if she's like this all the time.
- ☐ Avoid talking about suicide, because it might put the idea in Jeanie's head.
- ☐ Tell Jeanie I have noticed something seems wrong, and I want to make sure she is okay.
- ☐ Suggest Jeanie tell a health professional about her problems (e.g. a counsellor, GP, or psychologist).
- ☐ Suggest Jeanie tell an adult (other than a health professional) about her problems (e.g. a parent or teacher).
- ☐ Ask Jeanie if she is thinking of suicide.
- ☐ Encourage Jeanie to take responsibility and deal with her problems on her own.
- ☐ Listen to Jeanie talk about her problems.
- ☐ Not do anything.

Other (please specify):

15. If Jeanie was a friend, how confident would you feel in helping her?

Not at all confident	A little bit confident	Moderately confident	Quite a bit confident	Extremely confident
○	○	○	○	○

16. Which of the following people do you think would be helpful, harmful, or neither helpful nor harmful, for Jeanie’s problem?

	Helpful	Neither	Harmful
Counsellor	◯	◯	◯
General practitioner (GP) or family doctor	◯	◯	◯
Psychologist	◯	◯	◯
Close friend	◯	◯	◯
Family member	◯	◯	◯
Parent	◯	◯	◯
Teacher	◯	◯	◯
School Welfare Coordinator/School Counsellor	◯	◯	◯
Minister or priest	◯	◯	◯

17. In the list provided in the question above, which one person do you think is the most helpful for Jeanie's problem?

- ◯ Close friend
- ◯ Counsellor
- ◯ Family member
- ◯ General practitioner (GP) or family doctor
- ◯ Minister or priest
- ◯ Parent
- ◯ Psychologist
- ◯ School Welfare Coordinator/School Counsellor
- ◯ Teacher
- ◯ None of the above

18. If I had a problem right now like Jeanie's, I would...
Please note: you can select more than one option.

- [ ] Try to deal with it on my own.
- [ ] Talk to a friend about it.
- [ ] Talk to a health professional about it (e.g. a counsellor, GP or psychologist).
- [ ] Talk to an adult (other than a health professional) about it (e.g. a parent or teacher).
- [ ] Improve my diet.
- [ ] Try to get more sleep.
- [ ] Spend more time with friends.
- [ ] Try to do more exercise.
- [ ] Look for information about my problem on the internet.
- [ ] Do nothing.

Other (please specify)

19. Please indicate how strongly you personally agree or disagree with each statement

	Strongly disagree	Disagree	Neither agree nor disagree	Agree	Strongly agree
People with a problem like Jeanie's could snap out of it if they wanted.	○	○	○	○	○
A problem like Jeanie's is a sign of personal weakness.	○	○	○	○	○
Jeanie’s problem is not a real medical illness.	○	○	○	○	○
People with a problem like Jeanie's are dangerous.	○	○	○	○	○
It is best to avoid people with a problem like Jeanie's so that you don’t develop this problem.	○	○	○	○	○
People with a problem like Jeanie's are unpredictable.	○	○	○	○	○
If I had a problem like Jeanie's I would not tell anyone.	○	○	○	○	○

20. The following questions ask how you would feel about spending time with Jeanie.
Would you be happy to:

	Yes definitely	Yes probably	Probably not	Definitely not
Go out with Jeanie on the weekend?	◯	◯	◯	◯
Work on a project with Jeanie?	◯	◯	◯	◯
Invite Jeanie around to your house?	◯	◯	◯	◯
Go to Jeanie’s house?	◯	◯	◯	◯
Develop a close friendship with Jeanie?	◯	◯	◯	◯

teenAID - Experience of first aid situations

This page asks about your experiences of common physical problems requiring first aid. Please read each question carefully and fill out any boxes when they apply.

21. Since you completed the first aid course 12 months ago, have you come across someone*about your age (i.e. between 13-18 years old)* who has required first aid because of a physical emergency or injury?

Please do not report any adults you have had contact with. For this question, we are just interested in people around your age who you may have helped.

This question excludes first aid you have provided for mental health problems or crises.

◯ Yes - please go on to Q22 below.

◯ No - please go on to Q26 on the next page.

◯ Not sure - please go on to Q22 below.

◯ I don't want to answer this question - please go on to Q26 on the next page.

teenAID survey: help you have given

This page contains questions about the help you provided to the person/people about your age.

22. Please tell us *how many people about your age* you had contact with who required first aid for a physical injury or emergency:

[ ]

If you have had contact with **more than one** person about your age who required first aid, please answer the following questions ***about the person you know best.*** If you only had contact with one person, please answer the following questions about that person.

23. Did you offer the person any help?

- ◯ Yes - please go on to Q24 below.
- ◯ No - please go on to Q25 on the next page.
- ◯ Not sure - please go on to Q24 below.

teenAID: help you have given

This page asks about what you did to help the person.

24. What did you do to help the person?

teenAID: help you have given

This page contains questions about why you may not have been able to help the person.

25. What was the reason(s) that you were not able to help the person?

teenAID: help you have received

26. Since you completed the first aid course 12 months ago, have*you* required first aid because of a physical emergency or injury?

This question excludes first aid you have been given for mental health problems or crises.

- ◯ Yes - please go on to Q27 below.
- ◯ No - please go on to Q30 below.
- ◯ Not sure - please go on to Q27 below.
- ◯ I don't want to answer this question - please go on to Q30 below.

teenAID - Support for injury or first aid situation

This page contains questions about the support you have received for your physical injury or emergency.

27. Did you receive any first aid for this physical injury or emergency?

- ◯ Yes - please go on to Q28 below.
- ◯ No - please go on to Q30 on the next page.
- ◯ Not sure - please go on to Q28 below.

teenAID: help you have received

This page asks about who provided help to you for this emergency or injury.

28. Who provided first aid to you?
Please note: you can select more than one option.

- ☐ Friend (if you select this option, please also answer Q29 on the next page).
- ☐ Parent
- ☐ Other family member
- ☐ Medical or health professional (e.g., a GP, hospital staff, or physiotherapist)
- ☐ Teacher

Other (please specify)

____

teenAID: help you have received

This page asks about the help you received from your friend.

29. *Please only answer this question if you reported that a friend helped you in the previous question.*
Now think specifically about the help that *your friend* provided.
What did your friend do to help you?

____

teenAID - Experience of mental health problems

This page asks some questions about your experiences of mental health problems. Please read each question carefully and fill out any boxes when they apply.

DEFINITIONS
A mental health problem is when there is a major change in a person's normal way of thinking, feeling or behaving, which interferes with the person's ability to get on with life, and does not go away quickly or lasts longer than normal emotions or reactions would be expected to. A mental health problem might involve a diagnosed mental illness, a worsening of mental health, an undiagnosed problem, or a drug or alcohol problem.

A mental health crisis is when a person is at increased risk of harm to themselves or to others. Crisis situations include having thoughts of suicide, engaging in self-injury, being very intoxicated with alcohol or other drugs, or experiencing bullying or abuse.

30. Since you completed the first aid course 12 months ago, have you*had contact with anyone about your age (i.e. between 13-18 years old)* who you thought might have a mental health problem or has experienced a mental health crisis?

Please do not report any adults you have had contact with. For this question, we are just interested in people around your age who you may have helped.

- ○ Yes - please go on to Q31 below.
- ○ No - please go on to Q35 on the next page.
- ○ Not sure - please go on to Q31 below.
- ○ I don't want to answer this question - please go on to Q35 on the next page.

teenAID survey: help you have given

This page contains questions about the help you provided to the person/people about your age.

31. Please tell us *how many people about your age* you had contact with who you thought might have a mental health problem or experienced a mental health crisis:

[ ]

If you have had contact with more than one person about your age who you thought might have a mental health problem or experienced a mental health crisis, please answer the following questions ***about the person who you know best.*** If you only had contact with one person, please answer the following questions about that person.

32. Did you offer the person any help?

- ◯ Yes - please go on to Q33 below.
- ◯ No - please go on to Q34 below.
- ◯ Not sure - please go on to Q33 below.

teenAID: help you have given

This page contains questions about what you did to help the person.

33. What did you do to help the person?
Please note: you can select more than one option.

- ☐ Told them what they needed to do to fix their problems.
- ☐ Invited them to hang out and do something fun with me.
- ☐ Ignored them because they were being attention-seeking.
- ☐ Let them know I didn't want to be friends with them any more if they're like this all the time.
- ☐ Avoided talking about suicide, because it might have put the idea in their head.
- ☐ Told them I had noticed something seems wrong, and I wanted to make sure they were okay.
- ☐ Suggested they tell a health professional about their problems (e.g. a counsellor, GP, or psychologist).
- ☐ Suggested they tell an adult (other than a health professional) about their problems (e.g. a parent or teacher).
- ☐ Asked them if they were thinking of suicide.
- ☐ Encouraged them to take responsibility and deal with their problems on their own.
- ☐ Listened to them talk about their problems.
- ☐ I didn't do anything.

Other (please specify):

teenAID: help you have given

This page contains questions about why you may not have been able to help the person.

34. What was the reason(s) that you were not able to help the person?

teenAID survey: your own mental health

35. Since you completed the first aid course 12 months ago, have ***you*** had a mental health problem or experienced a mental health crisis?
Please report any mental health problem you think you might have experienced, whether or not it has been formally diagnosed by a doctor or other mental health professional.

- ◯ Yes - please go on to Q36 below.
- ◯ No - please go on to Q39 on the next page.
- ◯ Not sure - please go on to Q36 below.
- ◯ I don't want to answer this question - please go on to Q39 on the next page.

teenAID - Support for mental health problem

This page contains questions about the support you have received for your mental health problem.

36. In the last 12 months has anyone tried to support or assist you with this mental health problem or crisis?

- ◯ Yes - please go on to Q37 below.
- ◯ No - please go on to Q39 on the next page.
- ◯ Not sure - please go on to Q37 below.

teenAID: help you have received

This page contains questions about the person who helped you.

37. Who provided support or assistance for the problem?
Please note: you can select more than one option.

- ☐ Friend (if you select this option, please also answer Q38 on the next page).
- ☐ Parent
- ☐ Other family member
- ☐ Health professional (e.g. a counsellor, GP or psychologist)
- ☐ Teacher

Other (please specify)

[ ]

teenAID: help you have received

This page asks about the help you received from your friend.

38. *Please only answer this question if you reported that a friend helped you in the previous question*

Now think specifically about the help that *your friend* provided.
What did your friend do to help you?
Please note: you can select more than one option.

- [ ] Told me they had noticed something seems wrong, and wanted to make sure I was okay.
- [ ] Ignored me because they thought I was being attention-seeking.
- [ ] Asked me if I was thinking of suicide.
- [ ] Listened to me talk about my problems.
- [ ] Told me what I needed to do to fix my problems.
- [ ] Suggested I tell a health professional about my problems (e.g. a counsellor, GP, or psychologist).
- [ ] Suggested I tell an adult (other than a health professional) about my problems (e.g. a parent or teacher).
- [ ] Encouraged me to take responsibility and deal with my problems alone.
- [ ] Invited me to hang out and do something fun with them.
- [ ] Let me know they didn't want to be friends with me any more if I'm like this all the time.

Other (please specify):

teenAID - Final questions

39. What do you think the <u>strengths</u> of the first aid course were?

40. What do you think the <u>weaknesses</u> of the first aid course were?

41. How would you make the first aid course better?

42. Is there anything else you would like to say about the program?

teenAID - How you are feeling

This section contains questions about how you have been feeling. Please answer as truthfully as possible.

43. In the past 30 days...

	None of the time	A little of the time	Some of the time	Most of the time	All of the time
About how often did you feel nervous?	◯	◯	◯	◯	◯
About how often did you feel hopeless?	◯	◯	◯	◯	◯
About how often did you feel restless or fidgety?	◯	◯	◯	◯	◯
About how often did you feel so depressed that nothing could cheer you up?	◯	◯	◯	◯	◯
About how often did you feel that everything is an effort?	◯	◯	◯	◯	◯
About how often did you feel worthless?	◯	◯	◯	◯	◯

teenAID - Follow-up surveys

This section asks about future questionnaires relating to this research.

Follow-up surveys relating to this research project may be conducted at the school in future, but we would also like to have another way of contacting you in the event that you move or change schools.

44. Are you willing to be contacted by the research team about follow-up surveys related to this study?

◯ Yes

◯ No

45. If you are willing to be contacted about follow-up surveys related to this study, please enter your personal email address and a phone number that we can contact you on in the future.

Email address: ____________________

Phone number: ____________________

Please note that any personal information that you share with us will be stored securely, and your email and phone details will be stored separately from your other survey responses.

End Survey

That is the end of the survey. Thank you for your contribution!

If you are feeling distressed at any stage and would like some support, please try talking to your school welfare coordinator. Alternatively, you could try talking to your home room teacher or parents.

You could also try one of the following free services:

Kids Helpline: Phone 1800 55 1800 or visit kidshelp.com.au

Lifeline: Phone 13 11 14 or visit lifeline.org.au

ReachOut: Visit au.reachout.com

If you would like further information about this research, please contact
Ms. Alyssia Rossetto
email: alyssia.rossetto@unimelb.edu.au, phone: 03 9035 6029
Population Mental Health Group, University of Melbourne
Alyssia is the Research Assistant for the teenAID evaluation project.

If you have questions about the first aid training or first aid instructors, please contact the Research Assistant, Ms. Alyssia Rossetto, who can forward on your query to Australian Red Cross.

Laura Hart is the Chief Investigator of the teenAID evaluation project, and is responsible for the design and conduct of the trial.
email: lhart@unimelb.edu.au
Centre for Mental Health, University of Melbourne

If you have questions about the scientific aspects of the study, please contact:
Professor Tony Jorm
email: ajorm@unimelb.edu.au, phone: 03 9035 7799
Centre for Mental Health, University of Melbourne
Tony is a Professorial Fellow and Scientific Director of MHFA Australia.

This research has been approved by the University of Melbourne Human Research Ethics Committee.
If you have concerns about the ethics of the study, please contact:
Manager
Human Research Ethics, The University of Melbourne
phone: 03 8344 2073
Ethics approval number: 1341238.3
Version 2 May 2014
