# Supplementary material for: Frequency and quality of first aid offered by older adolescents: a cluster randomised crossover trial of school-based first aid courses
Source: PeerJ. 2020 Aug 17;8:e9782. doi: 10.7717/peerj.9782 (PMC7439956; doi:10.7717/peerj.9782)
Supplement: Supplemental Information 11 [file peerj-08-9782-s011.pdf]

ANZCTR
Australian New Zealand Clinical Trials Registry

CREATE ACCOUNT LOGIN

DEFINITIONS HINTS AND TIPS FAQs REGISTER TRIAL MY TRIALS

Trial Review

Please be advised that ANZCTR staff are prioritising submissions of COVID-19 trials occurring in Australia and New Zealand. As such, there are delays in reviews of non-COVID-19 submissions, international submissions, and updates.
Additionally, due to the ongoing COVID-19 pandemic, ANZCTR staff will **not** be able to answer the ANZCTR phone line.
Please email any inquiries to info@actr.org.au and we will respond as soon as possible.
Apologies for any inconvenience caused.

VIEW TRIAL AT REGISTRATION VIEW HISTORY

The safety and scientific validity of this study is the responsibility of the study sponsor and investigators. Listing a study does not mean it has been endorsed by the ANZCTR. Before participating in a study, talk to your health care provider and refer to this information for consumers

< BACK

Trial registered on ANZCTR

**Registration number**	ACTRN12614000061639
**Ethics application status**	Approved
**Date submitted**	22/11/2013
**Date registered**	20/01/2014
**Date last updated**	13/09/2017
**Type of registration**	Prospectively registered

Titles & IDs

**Public title**	teen Mental Health First Aid: A Cluster Randomised Controlled Trial
**Scientific title**	Cluster randomised controlled trial of secondary school student training in teen Mental Health First Aid versus physical first aid on ability to assist peers with a mental health problem.
**Secondary ID [1]**	Nil
**Universal Trial Number (UTN)**	U1111-1150-4287
**Trial acronym**	
**Linked study record**	

Health condition

Health condition(s) or problem(s) studied:

Adolescent Mental Health

Youth Suicide prevention

Condition category	Condition code
Mental Health	Suicide
Mental Health	Depression
Mental Health	Anxiety

Intervention/exposure

**Study type**	Interventional
**Description of intervention(s) / exposure**	teen Mental Health First Aid (teen MHFA) is a new training course for adolescents in the upper High School

years. The key messages of the program were developed using the expert consensus of youth mental health consumer advocates and MHFA instructors who work with youth. The program focuses on developing knowledge and skills in:
- recognising warning signs that a peer is developing a mental health problem
- understanding how to talk to a peer about mental health and seeking help
- when and how to tell a responsible adult
- where to find appropriate and helpful resources about mental illness and professional help
- how to respond in a crisis situation, including where a peer is suicidal or self-harming.

The teen MHFA Program consists of 3x 1 hour sessions of a teen MHFA training course for adolescents. These 3 sessions are spread over a maximum of 3 weeks (1 session p/week) depending on each school's timetabling requirements. Sessions are administered by an experienced Youth Mental Health instructor, with a student welfare staff member also present, to class groups of between 20-25.

Also included in the teen MHFA program is the offer of the 14-hour Youth MHFA course for teachers and parents of adolescents who attend the teen MHFA course. This is administered by the same experienced Youth Mental Health instructor, and can be given in either: 2 days with 7 hours of training, or 3-4 shorter sessions of an evening, depending on what suits each school community.

In the following year, a new cohort of Year 10 students will receive the cross-over (opposite) intervention, while the original cohort complete 12 month follow-up measures after the first intervention.

Intervention code [1] Prevention

Intervention code [2] Early detection / Screening

Comparator / control treatment The active control will be a 3-hour abbreviated Physical First Aid course. This will cover: what is first aid, chain of survival, DRSABCD, recovery position, CPR, defibrillation, and basic first aid for common sports injuries: sprains, strains, wound care esp. abrasions and nose bleeds, fractures and dislocations, concussion, and asthma.

The physical first aid course will also be administered in 3x 1hr sessions, over a period of around 5-8 schools days (depending on school timetabling). Sessions will be administered by an experienced Red Cross first aid instructor, in class groups of 20-25 students.

Control group Active

Outcomes

Primary outcome [1] First Aid intentions will be measured using forced choice responses to a vignette describing a young person with depression and suicidal thoughts. Participants will be asked to asked to select which of 12 possible actions they would take towards the person in the vignette. The possible actions are to engage in different first aid actions. Response options are based on messages that are either consistent with the teen MHFA Action Plan (6 items, e.g. “Ask John if he is thinking about suicide”) or distractor items that are contrary to the plan (6 items, e.g. “Avoid talking about suicide, because it might put the idea in John’s head”). Response items are scored as a criterion- referenced test against the action plan. Participants will be able to respond ‘other (please specify)’.

Timepoint [1] The outcome will be measured at three time points: baseline (before training begins), post-training (immediately after training) and follow-up (12 months after training).

Secondary outcome [1] First aid actions provided to a peer. This will be assessed using the first aid experiences questionnaire developed by Kitchener & Jorm for evaluations of Mental Health First Aid Training. It includes questions such "Have you come across someone you thought might have a mental health problem or has experienced a mental health crisis?" (Jorm, Kitchener, & Mugford, 2005).

Timepoint [1] At baseline (before training begins) and follow-up (12 months after training).

Secondary outcome [2] Confidence in supporting a peer. This will be assessed using a 5-point Likert-type scale where 1 = Not at all confident and 5 = Extremely confident.

Timepoint [2] The outcome will be measured at three time points: baseline (before training begins), post-training (immediately after training) and follow-up (12 months after training).

Eligibility

Key inclusion criteria Schools to be included in the trial must be high schools located in the greater Melbourne region. Year 10 students from these schools will be involved in the research. We estimate conservatively that there will be 100 Year 10 students per school.

Minimum age 15 Years

Maximum age 17 Years

Gender Both males and females

Can healthy volunteers participate? Yes

Key exclusion criteria teen Mental Health First Aid is not designed as a suicide postvention program. If a recent suicide has occured in a school, the appropriateness of running the teen MHFA program will be assessed before formally recruiting the school.

Study design

Purpose of the study Prevention

Allocation to intervention Randomised controlled trial

Procedure for enrolling a subject and allocating the treatment (allocation concealment procedures) The design of the study is a cluster randomised controlled trial, using schools as the cluster. All schools will receive both teen Mental Health First Aid (the intervention) and physical first aid (the control), as the study employs a crossover design. See below for information about the order of training. In the first year of training, two schools will be randomly assigned to either the intervention or control using an online random sequence generator. In the following year, these schools will cross-over to receive the alternate intervention. The same procedure will be used for the remaining two schools in Year 2. Neither the schools nor the instructors will be blinded to allocation as they will be aware which training they are receiving and in what order. However, researchers handling the final dataset and coding participant responses will be blinded to group and time variables.

Allocation is not concealed because the person who determines the random allocation is also likely to know whether or not the school (cluster) is eligible to participate.

Methods used to generate the sequence in which subjects will be randomised (sequence generation) The four schools will be randomised by using an online random number sequence generator.
Schools will be assigned a number (from 1-4) based on the order in which the enrol in the study. A random sequence of 1-4 will be generated, which will determine the order in which the schools are provided the intervention. Schools will receive both courses over two years; randomisation will determine the order in which schools receive the intervention.

Masking / blinding Blinded (masking used)

Who is / are masked / blinded?

The people analysing the results/data

Intervention assignment Crossover

Other design features

Phase Not Applicable

Type of endpoint(s) Safety/efficacy

Statistical methods / analysis Data analysis for this study will include descriptive statistics, such as means and percentages, to examine the main socio-demographic characteristics of the students.

The hypotheses will be analyzed using mixed models for continuous and binary outcome variables, with group-by-measurement-occasion interactions. This method of analysis is well suited to the data that will be collected for this study as it takes into account its hierarchical structure, i.e. the correlation of measurement occasions within students. While we expect the school year cohort cluster level to have little substantial impact on the results, adjustment for the correlation of student responses within school year cohort clusters will be made in the analyses of
the study. Another advantage of these maximum likelihood-based methods is that they are able to produce unbiased estimates when a proportion of the participants withdraw before the completion of the study, based on the reasonable assumption that these data are missing at random.

Possible effects of the order of school intervention and control conditions on the outcomes of interest will also be examined – i.e., whether school year cohorts that received the intervention first differed in their responses to those that received it after the control condition.

We estimate conservatively that there will be 200 Year 10 students per school and that half the parents will give consent for their child to participate. With 4 schools participating over 2 years, this gives 8 clusters and 400 adolescents. Given the matching of schools and counter-balancing of school conditions in the study design, and our previous research (Jorm, Kitchener, Sawyer, Scales, Cvetkovski, 2010) which showed a very small school cluster influence on the intra-class correlation for students (ICC=0.003), we have ignored the school cluster level in these power calculations. According to Stata 12 software, with 400 students (200 per intervention and control), and assuming a 0.70 correlation between pre-post measurements, the study will have a 0.80 power to detect small (d=0.17) group-by-measurement occasion differences in the outcomes of interest at an alpha level of 0.05. Even with a further loss of up to 20% of students due to attrition (320, 160 per intervention and control), the study will still have a 0.80 power to detect a small effect size (d=0.19) at an alpha level of 0.05.

Recruitment

Recruitment status Completed

Date of first participant enrolment

Anticipated 3/03/2014 **Actual** 2/06/2014

Date of last participant enrolment

Anticipated **Actual** 10/08/2016

Date of last data collection

**Anticipated**	23/08/2017	**Actual**	17/08/2017

Sample size

**Target**	400	**Accrual to date**		**Final**	1938

Recruitment in Australia

Recruitment state(s) VIC

Funding & Sponsors

**Funding source category [1]**	Charities/Societies/Foundations
**Name [1]**	Australian Rotary Health
**Address [1]**	PO Box 3455<br>Parramatta, NSW 2124
**Country [1]**	Australia
**Funding source category [2]**	Government body
**Name [2]**	National Health and Medical Research Council
**Address [2]**	16 Marcus Clarke St<br>Canberra, ACT 2601
**Country [2]**	Australia
**Primary sponsor type**	University
**Name**	University of Melbourne
**Address**	The University of Melbourne<br>Victoria 3010
**Country**	Australia
**Secondary sponsor category [1]**	Charities/Societies/Foundations
**Name [1]**	Mental Health First Aid Australia
**Address [1]**	Level 6, 369 Royal Parade<br>Parkville VIC 3052
**Country [1]**	Australia

Ethics approval

**Ethics application status**	Approved
**Ethics committee name [1]**	University of Melbourne Human Ethics Advisory Group (HEAG)
**Ethics committee address [1]**	207 Bouverie St<br>The University of Melbourne<br>Parkville VIC 3010
**Ethics committee country [1]**	Australia
**Date submitted for ethics approval [1]**	05/12/2013
**Approval date [1]**	11/02/2014
**Ethics approval number [1]**	
**Ethics committee name [2]**	University of Melbourne Human Ethics Sub-Committee (Health Sciences HESC)
**Ethics committee address [2]**	Human Research Ethics<br>Office for Research Ethics and Integrity<br>Level 1, 780 Elizabeth Street (near cnr Grattan Street), (University Building No. 220)<br>Melbourne VIC 3010
**Ethics committee country [2]**	Australia
**Date submitted for ethics approval [2]**	03/02/2014
**Approval date [2]**	18/03/2014
**Ethics approval number [2]**	
**Ethics committee name [3]**	Department of Education and Early Childhood Development
**Ethics committee address [3]**	2 Treasury Place<br>East Melbourne<br>Victoria<br>3002
**Ethics committee country [3]**	Australia

Date submitted for ethics approval [3] 18/04/2014

Approval date [3] 15/04/2014

Ethics approval number [3]

Summary

Brief summary

Mental health problems frequently have their first onset during adolescence, but adolescents often do not seek professional help or show long delays in getting professional help. They show a strong preference to seek help from informal sources such as family and friends. However, friends may lack the knowledge and skills to provide effective support.

teen MHFA training is designed to give adolescents the basic skills they need to support peers and connect them with an adult helper. The aim of this trial is to compare the effects of teen MHFA training of Year 10 students with a physical first aid training control group on: recognition of mental disorders, help-seeking beliefs, mental health first aid intentions, stigmatising attitudes, confidence in supporting a peer, help provided to a peer, and mental health. In addition, the trial aims to provide initial evidence on whether teen MHFA training could have potential as a suicide prevention program by improving ability of students to assist a suicidal peer.

Trial website

Trial related presentations / publications

Ross AM, Hart LM, Jorm AF, Kelly CM, Kitchener BK. Development of key messages for adolescents on pro viding basic mental health first aid to peers: a delphi consensus study. Early Intervention in Psychiatry 2012; 6:229–238.

Public notes

Contacts

Principal investigator

Name Dr Laura Hart

Address University of Melbourne,
207 Bouverie St, Carlton VIC 3010

Country Australia

Phone +61 3 9035 6771

Fax

Email lhart@unimelb.edu.au

Contact person for public queries

Name Ms Alyssia Rossetto

Address University of Melbourne,
207 Bouverie St, Carlton VIC 3010

Country Australia

Phone +61 3 9035 6029

Fax

Email alyssia.rossetto@unimelb.edu.au

Contact person for scientific queries

Name Dr Laura Hart

Address University of Melbourne,
207 Bouverie St, Carlton VIC 3010

Country Australia

Phone +61 3 9035 6771

Fax

Email lhart@unimelb.edu.au

No information has been provided regarding IPD availability

Summary results

Have study results been published in a peer-reviewed journal?

Other publications

Have study results been made publicly available in another format?

Results – basic reporting

Results – plain English summary

< BACK

ANZCTR

Home
About us
Statistics
Useful links
News
Contact
Privacy
Terms and conditions

Register a trial

Create account
Login
How to register a trial
How to update a trial
Data item definitions
Hints and tips
FAQs

Search for a trial

Find a trial
How to search
How to get involved

Major funders

Privacy | Disclaimer
Web design by G Squared

Copyright © Australian New Zealand Clinical Trials Registry. All rights reserved.
